# Impacts of *Mir146a* Genotypes on Bladder Cancer Risk in Taiwan

**DOI:** 10.3390/biomedicines11051396

**Published:** 2023-05-08

**Authors:** Bo-Ren Wang, Wen-Shin Chang, Cheng-Hsi Liao, Yun-Chi Wang, Jian Gu, Da-Tian Bau, Chia-Wen Tsai

**Affiliations:** 1 Graduate Institute of Biomedical Sciences, China Medical University, Taichung 404333, Taiwan; 2 Division of Urology, Department of Surgery, Taichung Armed Forces General Hospital, Taichung 41152, Taiwan; 3 National Defense Medical Center, Taipei 11490, Taiwan; 4 Terry Fox Cancer Research Laboratory, Department of Medical Research, China Medical University Hospital, Taichung 404332, Taiwan; 5 Department of Epidemiology, The University of Texas MD Anderson Cancer Center, Houston, TX 77030, USA; 6 Department of Bioinformatics and Medical Engineering, Asia University, Taichung 41354, Taiwan

**Keywords:** bladder cancer, genotype, *mir146a*, *mir196a*, phenotype, single-nucleotide polymorphism

## Abstract

The aim of this study was to investigate the association between single-nucleotide polymorphisms (SNPs) in *mir146a* and *mir196a* and bladder cancer (BLCA) risk in Taiwan. The genotypes of *mir146a* rs2910164 and *mir196a* rs11614913 were determined in 375 BLCA patients and 375 healthy controls using PCR-RFLP methodology, and their associations with BLCA risk were evaluated. The study also measured the serum expression level of *mir146a* using quantitative RT-PCR. The results showed that the distributions of CC, CG and GG genotypes of *mir146a* rs2910164 were 31.7%, 45.6% and 22.7% in the control group, and 21.9%, 44.3% and 33.8% in the case group, respectively. In logistic regression analyses, the heterozygous variant genotype CG carriers showed a marginally significant association with increased BLCA risk (OR = 1.41, 95% CI = 0.99–2.01), while the homozygous variant genotype GG carriers had a 2.17-fold increased risk of BLCA (OR = 2.17, 95%CI = 1.46–3.21). Moreover, carriers of the GG/CG genotypes had significantly higher serum levels of *mir146a* than those with the CC genotype (*p* < 0.0001), indicating a genotype–phenotype correlation. In contrast, *mir196a* rs11614913 was not associated with BLCA risk. Therefore, the genotypes of *mir146a* rs2910164 may serve as a useful biomarker for predicting the risk of BLCA.

## 1. Introduction

Bladder cancer (BLCA) is the fourth and eleventh most prevalent cancer among global males and females, respectively [1]. The incidence of BLCA ranks eleventh and sixteenth among cancers in Taiwan for males and females, respectively, and continues to rise [2]. The male-to-female BLCA ratio is approximately 5:2, with smoking habits being the reason for male predominance [3]. The etiology of BLCA may be due to complex interactions between environmental and genetic factors. Smoking is the predominant risk factor, and exposure to chemicals and PM2.5, prior radiation therapy, as well as frequent bladder infections, may also be involved [4,5]. Several studies have also identified meat as a possible risk factor for BLCA, and the associations between meat intake and BLCA may depend on the type of meat consumed, the cooking method and the temperature used [6]. On the other hand, although a family history of cancer provides strong support for the role of inheritance in BLCA development, the contribution of genetic factors and the underlying mechanisms remains largely unrevealed.

In the literature, accumulated studies have reported urine biomarkers to serve as predictors for BLCA, but they are still far from satisfying [7]. In addition, comprehensive screening methodologies have been developed in urine, taking advantage of its non-invasiveness to detect cell-free DNAs, cell-free RNAs, small RNAs, and DNA methylation status [8]. Noticeably, some alterations in circulating mRNAs, such as IGFBP5, HOXA13, MDK, CDK1, and CXCR2 [9], have been found to be potentially practicable. However, the diagnostic efficacy of these early BLCA markers remains insufficient since the sensitivity and specificity of each marker vary greatly among populations. Therefore, the development of useful biomarkers for BLCA risk and early detection remains an urgent need.

MicroRNAs (miRNAs) are a group of non-coding and single-stranded RNAs that act as negative regulators of gene expression [10]. Although they represent only 1 to 4% of genes in the human genome, a single miRNA can regulate approximately 200 target mRNAs, and we are just beginning to understand the network among them [11]. MiRNAs play critical roles in diverse biological functions, including cell proliferation, tissue remodeling, embryonic development, apoptosis, and most importantly, carcinogenesis [11,12]. Genetic variations in miRNA genes, along with their target genes, have been observed in a variety of human diseases, and the accumulated literature has suggested that impaired miRNAs may play a critical role during tumorigenesis [13,14]. RNA is much more vulnerable than DNA. However, as circulating miRNAs are packaged in exosome particles, they are protected from RNase degradation. These stable, circulating miRNAs could act as noninvasive biomarkers reflecting certain physiological statuses, including cancer [15,16]. More recently, Lin and Tsai reported that the expression levels of *mir146a* and *mir196a* were significantly increased in bladder tumors and urines from BLCA patients [17].

SNP is the most common type of genetic variation. SNPs in miRNA genes may alter the expression and function of specific miRNAs, which may contribute to the processes of tumorigenesis [18]. Several studies have analyzed the relationship between common SNPs in miRNA genes and BLCA risk [19,20,21,22]. However, no studies have evaluated the associations between miRNA SNPs and BLCA risk in Taiwan.

**Figure 1:**
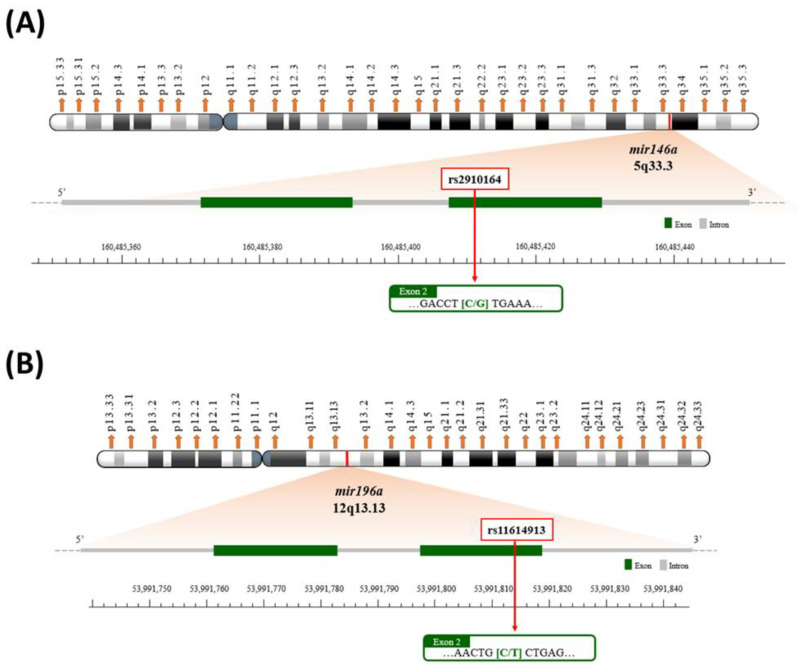
Map of *mir146a* rs2910164 (**A**) and *mir196a* rs11614913 (**B**) polymorphic sites.

In this study, our aim was to examine the associations between the two most widely studied miRNA SNPs, *mir146a* rs2910164 and *mir196a* rs11614913 (the map of polymorphic sites shown in Figure 1), with the risk of BLCA in Taiwan. In addition, we aimed to evaluate the interactions of miRNA genotypes and age, gender, smoking and alcohol drinking status on BLCA risk. Furthermore, we explored the genotype–phenotype correlation.

## 2. Materials and Methods

### 2.1. Recruitment of Bladder Cancer Patients and Healthy Controls 

The study was approved by the Institutional Review Board of China Medical University Hospital (DMR104-IRB-158). All participants are Taiwanese and have completed written informed consents. All the protocols are performed according to the principles of the Declaration of Helsinki. The inclusion and exclusion criteria have been defined as previously published [23]. Several demographic characteristics of all the cases and controls are concisely summarized in Table 1.

### 2.2. Genotyping Methodology of Mir146a and Mir196a SNPs and Quality Control

Genomic DNA was extracted from the blood samples using a Qiagen kit (Qiagen, Chatsworth, CA, USA) as usual [24]. The genotyping for *mir146a* rs2910164 and *mir196a* rs11614913 was conducted using the polymerase chain reaction restriction fragment length polymorphism (PCR-RFLP) methodology. For *mir146a* rs2910164, the PCR-RFLP genotyping was conducted with forward and reverse primers of 5′-CATGGGTTGTGTCAGTGTCAGAGCT-3′ and 5′-TGCCTTCTGTCTCCAGTCTTCCAA-3′, respectively. The genotyping of *mir196a* rs11614913 was performed with forward and reverse primers of 5′-CCCCTTCCCTTCTCCTCCAGATA-3′ and 5′-CGAAAACCGACTGATGTAACTCCG-3′, respectively. The PCR was carried out in a PCR Thermocycler (Bio-RAD, Hercules, CA, USA) using the following conditions: initial denaturation at 94 °C for 5 min, followed by denaturation at 94 °C for 30 s, annealing at 64 °C for 40 s, and extension at 72 °C for 45 s. After 35 repeated PCR cycles, a final extension step was conducted at 72 °C for 10 min. PCR amplicons for *mir146a* and *mir196a* were identified via 3% agarose gel electrophoresis to confirm the success of the original PCR, and then cut by *Sac* I and *Msp* I, respectively, followed by reconfirmation using 4% agarose gel. The *mir146a* SNP presented three different patterns: an intact single 147 bp fragment for the GG genotype; fully digested fragments of 122 and 25 bp for the CC genotype; and fragments of 147, 122, and 25 bp for the heterozygous GC genotype [25]. The *mir196a* SNP presented 3 different patterns: an intact single 149 bp fragment for the TT genotype; full-digested fragments of 125 and 24 bp for the homologous variant CC genotype; and fragments of 149, 125, and 24 bp for the heterozygous variant CT genotype, respectively [26]. For quality control, we validated the PCR-RFLP genotype results by sequencing 6 random samples with different representative genotypes. We also randomly selected 36 (4.8%) samples from both the control (n = 18) and case (n = 18) groups for repeat genotyping by an independent technician, and the genotypes were 100% concordant. All the lab personnel performing genotyping were blinded to the case–control statuses.

### 2.3. Quantitative Reverse Transcription Polymerase Chain Reaction for Measuring Mir146a Expression

According to the manufacturer’s manual, miRNA was first extracted from serum using the miRNeasy Mini Isolation kit (Qiagen, Redwood, CA, USA). These miRNA samples were then served as templates to synthesize complementary DNA (cDNA) using the miScript II RT kit (Qiagen, Redwood, CA, USA). This reverse transcription (RT) reaction was carried out under the following conditions: 42 °C for 15 min; 85 °C for 5 s; and then held at 4 °C. After the RT reaction, the cDNA products were diluted at a 1:100 ratio, and 1 μL of the diluted cDNA was used for subsequent quantitative RT-PCR. Mir146a expression was quantitated using the miScript SYBR Green PCR kit (Qiagen, Redwood, CA, USA) according to the manufacturer’s instructions. The primers were part of the SYBR green assay for *mir146a*. The small nuclear RNA U6 was used as a loading control for further normalization.

### 2.4. Statistical Methodology

The unpaired Student’s *t*-test was used to compare the ages (continuous variable) between the case and control groups. The Pearson’s chi-square test was used to compare the distributions of gender, personal habits, and different genotypes and alleles among those subgroups. The associations between different genotypes and bladder cancer risk were estimated with individual odds ratios (ORs) plus 95% confidence intervals (CIs). A trend test was used to evaluate the presence of a linear trend in cancer risk associated with increasing numbers of risk alleles in the additive model. Any result with a *p*-value less than 0.05 is taken as statistically significant.

## 3. Results

### 3.1. Demographic and Clinical Characteristics of Cases and Controls

Table 1 shows the demographic characteristics, including age, gender, and personal habits for all the participants, as well as the stage and grade of the 375 bladder cancer cases. Since we adopted a matching strategy for variables including age, gender, smoking and alcohol drinking status when recruiting healthy controls, there was no significant difference for those variables between the two groups (all *p* > 0.05). The male/female ratio among bladder cancer cases is approximately 3:1 (Table 1). The proportion of non-muscle-invasive and muscle-invasive types is 62.7% and 37.3%, respectively; while the proportion of low and high grades is 40.3% and 59.7%, respectively (Table 1).

### 3.2. Associations of Mir146a and Mir196a Genotypes with Bladder Cancer Risk

**Figure 2:**
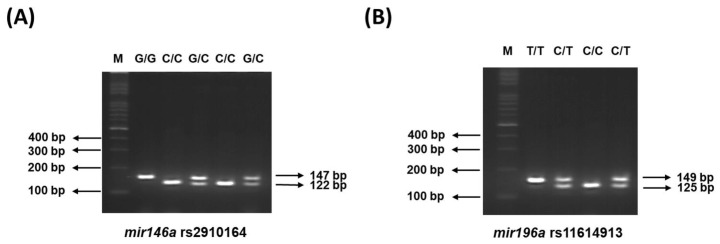
These are the representative plots of the polymerase chain reaction (PCR)–restriction fragment length polymorphism (RFLP) analyses of the (**A**) *mir146a* rs2910164 and (**B**) *mir196a* rs11614913, respectively. To detect polymorphisms via PCR and enzyme digestion, PCR products were fully digested or undigested with the corresponding restriction enzymes (*Sac* I and *Msp* I). The patients with homozygote wild type, heterozygote, and homozygote variant types are illustrated, and the genotypes are indicated.

First, the genotypes of *mir146a* rs2910164 and *mir196a* rs11614913 (as shown in Figure 2) in the control groups were consistent with the frequencies expected under the Hardy–Weinberg equation (both *p* > 0.05). Second, a significant association was found between *mir146a* rs2910164 SNP and BLCA risk in an additive model: individuals carrying the heterozygous variant genotype CG had a marginally significant increased BLCA risk (OR = 1.41, 95% CI = 0.99–2.01, *p* = 0.0695), while those carrying the homozygous variant GG had a 2.17-fold increased BLCA risk (OR = 2.17, 95% CI = 1.46–3.21, *p* = 0.0002) (*p* for trend = 0.0005). Individuals carrying variant genotypes (CG + GG) were at a 1.66-fold increased BLCA risk (OR = 1.66, 95% CI = 1.20–2.30, *p* = 0.0030) (Table 2). Regarding *mir196a* rs11614913, no significant association with BLCA risk was observed (Table 3).

### 3.3. Associations of Mir146a rs2910164 and Mir196a rs11614913 Alleles with BLCA Risk

The findings in Table 2 and Table 3 have been validated by analyzing the distributions of alleles at *mir146a* rs2910164 and *mir196a* rs11614913 in the BLCA and healthy control groups (Table 4). In detail, the allelic frequencies of the *mir146a* rs2910164 G allele of the controls were 45.5%, much lower than 56.0% in the BLCA patient group (OR = 1.53, 95% CI = 1.25–1.87, *p* = 0.0001) (Table 4). As for *mir196a* rs11614913, there was no such difference between the BLCA and healthy control groups (OR = 0.94, 95% CI = 0.76–1.15, *p* = 0.5667) (Table 4).

### 3.4. Stratified Analyses of Mir146a Genotypes by Age, Gender, Smoking, and Alcohol Drinking Status

We then performed stratified analyses of *mir146a* rs2910164 with BLCA risk by age, gender, smoking, and alcohol drinking status (Table 5). In general, the associations of *mir146a* rs2910164 SNP with BLCA risks were consistently significant in all the strata except for females, in which the risk for GG (OR = 2.01, 95% CI = 0.92–3.95) did not reach statistical significance, likely due to the small sample size.

### 3.5. Serum Expression Level of mir146a and Its Correlation with mir146a Genotypes

**Figure 3:**
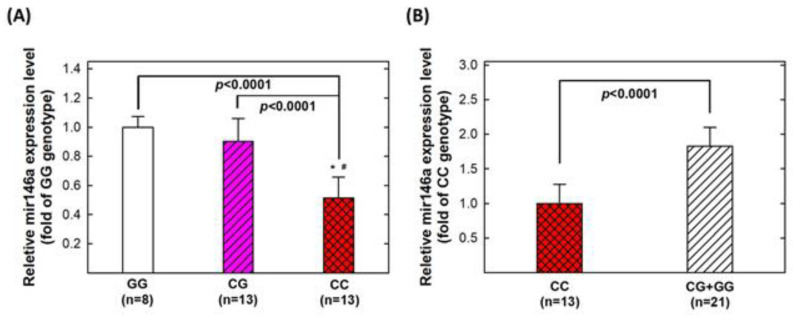
Correlation between *mir146a* genotype and mir146 expression in the serum of healthy subjects. (**A**) healthy people of the GG, CG and CC genotypes were shown independently; (**B**) those of the CG and GG were combined, and compared with CC genotype.* Significantly different from GG genotypes; # significantly different from CG genotypes.

We were interested in the genotype–phenotype correlation, and their contribution to BLCA risk. Thus, 34 serum samples of healthy controls were examined. The results showed that 13, 13, and 8 subjects were of CC, CG, and GG genotypes at *mir146a* rs2910164, respectively. Those people with the variant genotypes (GC and GG) had significantly higher levels of serum *mir146a* than those with the wild-type CC genotype (*p* < 0.0001) (Figure 3).

## 4. Discussion

In recent years, numerous studies have investigated the associations between SNPs in miRNA genes and the risks of various types of cancer [25,26,27,28,29,30]. Although conflicting and inconsistent results have been obtained, the differences in association among studies may be due to different dietary habits, environmental exposures, and most importantly, different genetic background among the various study populations.

In the current study, the frequencies of *mir146a* rs2910164 GG genotype and G allele were significantly higher in the BLCA patient group than the control group (Table 2 and Table 4). People with the *mir146a* rs2910164 GG genotypes had a 2.17-fold elevated risk of developing BLCA in Taiwan (Table 2). Four previous studies have assessed the associations of *mir146a* rs2910164 SNP with BLCA risk, with one in US, one in India, and two in mainland China [19,20,21,22]. Our study is the first study in Taiwan, and our results are consistent with the largest study to date in China, in which Wang et al. found that the *mir146a* rs2910164 C allele was associated with a decreased BLCA risk among 1019 Chinese BLCA cases and 1182 controls [20]. A meta-analysis of the four published studies showed that the C allele of rs2910164 is protective against bladder cancer (OR = 0.77, 95% CI = 0.68–0.88) [31]. These data strongly support the notion that the G allele of *mir146a* rs2910164 SNP is a BLCA risk allele.

Mir146a has been shown to play either an oncogenic or tumor suppressor role, depending on the cancer type and cellular context. The data from our study and previous reports strongly support an oncogenic role for *mir146a* in bladder cancer. It has been consistently shown that *mir146a* is upregulated in the bladder tumors and urine of BLCA patients in different studies [17,32,33]. In addition, Sasaki et al. [32] showed that BLCA patients with high-grade tumors exhibited significantly higher urinary miR-146a levels than those with low-grade tumors, and the patients with invasive tumors tended to show higher urinary miR-146a levels than those with noninvasive tumors. Furthermore, elevated urinary miR-146a levels in BLCA patients were decreased to the normal level after transurethral resection of the tumors. Functionally, Wang et al. [33] recently demonstrated that overexpression of *mir146a* promoted the invasion, migration, and proliferation of bladder cancer cell lines HT-1197 and HT-1376, supporting the oncogenic role of *mir146a* in vitro. All of these data support that *mir146a* acts as an oncomir in bladder cancer. In the current study, we found that individuals with risk-enhancing genotypes (CG and GG) at *mir146a* rs2910164 have significantly higher levels of serum *mir146a* (Figure 3). Together with the increased expression of *mir146a* in BLCA urines and tissues, these data further support the oncogenic role of *mir146a* in BLCA. Increased *mir146a*, as conferred by the variant allele G, increases BLCA risk. Furthermore, Wang et al. [20] found that the G allele of *mir146a* rs2910164 also conferred a significantly increased risk of BLCA recurrence, suggesting it may represent a biomarker for both risk prevention and therapeutic intervention.

The molecular mechanisms responsible for the oncogenic role of *mir146a* in bladder cancer are not yet fully understood. Previous studies have identified many genes that are important for DNA repair and that have tumor suppressor activity, and which are targeted directly by *mir146a*. These genes include DDIT3 (DNA damage-inducible transcript 3) [34], FANCM (Fanconi anemia, complementation group M) [35], Merlin tumor suppressor [36,37], NME1 (NME/NM23 nucleoside diphosphate kinase 1) [38], SMAD4 [39,40]. FLAP (5-Lipoxygenase Activating Protein) [41], HTT (Huntingtin) [42], CADM2 (cell adhesion molecule 2) [43], IRAK1 (interleukin 1 receptor associated kinase 1), TRAF6 (tumor necrosis factor receptor-associated factor-6), and NUMB (NUMB Endocytic Adaptor Protein) [44]. Which of these genes mediate the oncogenic effect of *mir146a* in bladder cancer warrants further study.

The results of our study did not reveal a significant association between *mir196a* rs11614913 and BLCA risk. This finding is consistent with a recent meta-analysis by Aziz et al. [45], who showed that although *mir196a* rs11614913 is associated with a significantly reduced risk of overall cancer, it is not associated with BLCA risk (OR = 0.90, 95% CI = 0.71–1.13). This meta-analysis included three BLCA studies, and all showed null results in an additive model [21,22,46]. Additionally, an earlier study in India [19] did not find a significant association in an additive model either. Overall, the literature suggests that *mir196a* rs11614913 is not associated with BLCA risk. 

There are some limitations to this study. Firstly, we were unable to perform analyses on the associations of these SNPs with prognosis due to patient heterogeneity. Prognosis analyses are ideally performed in homogeneously treated patients with similar clinical features. Future large studies are needed to address the prognostic values of these miRNA SNPs in Taiwan BLCA patients. Secondly, this study is a single-center study and is the only study performed to date in Taiwan. However, our study serves as a validation for other published studies. Our results require validation from other independent patient cohorts in Taiwan. Lastly, we did not collect diet data, and our cancer family history data were incomplete. Some potential confounding factors were not included in the analyses, which may have reduced the reliability of the results.

In summary, this study shows that the G allele and GG genotype of *mir146a* rs2910164 are associated with an increased risk of BLCA in Taiwanese people and are correlated with higher serum levels of *mir146a*. These findings suggest that the genotypes of *mir146a* rs2910164 could serve as a useful biomarker for predicting the risk of BLCA, and that serum *mir146a* expression levels could be a potential biomarker for the early detection of BLCA.

## Figures and Tables

**Table 1:** Selected demographic characteristics of the 375 BLCA patients and 375 non-cancer controls.

Character	Controls (*n* = 375)			Cases (*n* = 375)			*p*-Value
	*n*	%	Mean (SD)	*n*	%	Mean (SD)	
Age (years)			62.9 (9.8)			61.4 (10.3)	0.7315 ^a^
Age group (years)							0.7108 ^b^
≤55	152	40.5%		158	42.1%		
>55	223	59.5%		217	57.9%		
Gender							0.5525 ^b^
Male	287	76.5%		279	74.4%		
Female	88	23.5%		96	25.6%		
Personal habits							
Cigarette smoking	186	49.6%		201	53.6%		0.3063 ^b^
Alcohol drinking	176	46.9%		189	50.4%		0.3807 ^b^
Stage							
Non-muscle-invasive				235	62.7%		
Muscle-invasive				140	37.3%		
Grade							
Low				151	40.3%		
High				224	59.7%		

SD: Standard deviation; ^a^ based on Student’s *t*-test; ^b^ based on chi-square test.

**Table 2:** Distributions of *mir146a* rs2910164 genotypes in BLCA patient and control groups and the association of rs2910164 genotypes with BLCA risk.

SNP	Genotype	Cases	Controls	*p*-Value	OR (95% CI)
rs2910164	CC	82 (21.9%)	119 (31.7%)		1.00 (Ref)
	CG	166 (44.3%)	171 (45.6%)	0.0695	1.41 (0.99–2.01)
	GG	127 (33.8%)	85 (22.7%)	0.0002 *	2.17 (1.46–3.21)
*P*_trend_				0.0005 *	
	CG + GG	293 (78.1%)	256 (68.3%)	0.0030 *	1.66 (1.20–2.30)
*P*_HWE_				0.1193	

OR: odds ratio; CI: confidence interval; *p*-values for genotypes were calculated using the chi-square test with Yates’ correction; HWE: Hardy–Weinberg equilibrium; *P*_trend_, *p*-value for trend analysis; *P*_HWE_, *p*-value for Hardy–Weinberg equilibrium analysis *: *p* < 0.05.

**Table 3:** Distributions of *mir196a* rs11614913 genotypes in BLCA patient and control groups and the association of rs11614913 genotypes with BLCA risk.

SNP	Genotype	Cases	Controls	*p*-Value	OR (95%CI)
rs11614913	TT	125 (33.3%)	116 (30.9%)		1.00 (Ref)
	CT	180 (48.0%)	186 (49.6%)	0.5722	0.90 (0.65–1.24)
	CC	70 (18.7%)	73 (19.5%)	0.6549	0.89 (0.59–1.35)
*P*_trend_				0.7798	
	CT + CC	250 (66.7%)	259 (69.1%)	0.5316	0.90 (0.66–1.22)
*P*_HWE_				0.9195	

OR: Odds ratio; CI: confidence interval; *p*-Values for genotypes were calculated by the chi-square test with Yates’ correction; HWE: Hardy–Weinberg Equilibrium; *P*_trend_, *p*-value for trend analysis; *P*_HWE_, *p*-value for Hardy–Weinberg equilibrium analysis.

**Table 4:** Distributions of *mir146a* rs2910164 and *mir196a* rs11614913 alleles in BLCA patient and control groups and the associations of alleles with BLCA risk.

Allele	Cases	Controls	*p*-Value	OR (95% CI)
*mir146a* rs2910164				
C	330 (44.0%)	409 (54.5%)		1.00 (Ref)
G	420 (56.0%)	341 (45.5%)	0.0001 *	1.53 (1.25–1.87)
*mir196a* rs11614913				
T	430 (57.3%)	418 (55.7%)		1.00 (Ref)
C	320 (42.7%)	332 (44.3%)	0.5667	0.94 (0.76–1.15)

*p*-value was calculated using the chi-square test with Yates’ correction; *: *p* < 0.05.

**Table 5:** The associations of *mir146a* rs2910164 SNP with BLCA risk stratified by age, gender, smoking, and alcohol drinking status.

Genotype	Controls	Cases	OR (95% CI) ^a^	aOR (95% CI) ^b^	*p*-Value
Age					
≤55 years old					
CC	47	33	1.00 (ref)	1.00 (ref)	
CG	66	68	1.47 (0.84–2.57)	1.55 (0.87–2.34)	0.2283
GG	39	57	2.08 (1.14–3.81)	2.27 (1.18–3.56)	0.0248 *
>55 years old					
CC	72	49	1.00 (ref)	1.00 (ref)	
CG	105	98	1.37 (0.87–2.16)	1.49 (0.89–2.03)	0.2130
GG	46	70	2.24 (1.33–3.76)	2.58 (1.45–3.46)	0.0034 *
Gender					
Males					
CC	89	60	1.00 (ref)	1.00 (ref)	
CG	134	122	1.35 (0.90–2.03)	1.39 (0.87–1.96)	0.1810
GG	64	97	2.25 (1.43–3.54)	2.36 (1.66–3.31)	0.0007 *
Females					
CC	30	22	1.00 (ref)	1.00 (ref)	
CG	37	44	1.62 (0.80–3.27)	1.59 (0.77–3.18)	0.2402
GG	21	30	1.95 (0.89–4.26)	2.01 (0.92–3.95)	0.1391
Smoking behaviors					
Non-smokers					
CC	61	37	1.00 (ref)	1.00 (ref)	
CG	84	80	1.57 (0.94–2.62)	1.65 (0.97–2.47)	0.1077
GG	44	57	2.14 (1.21–3.77)	2.31 (1.33–2.98)	0.0125 *
Smokers					
CC	58	45	1.00 (ref)	1.00 (ref)	
CG	87	86	1.27 (0.78–2.08)	1.35 (0.83–2.03)	0.3985
GG	41	70	2.20 (1.27–3.81)	2.26 (1.32–3.74)	0.0069 *
Alcohol drinking behaviors					
Non-drinkers					
CC	61	41	1.00 (ref)	1.00 (ref)	
CG	95	79	1.24 (0.75–2.03)	1.32 (0.84–2.01)	0.4738
GG	43	66	2.28 (1.32–3.96)	2.34 (1.37–4.04)	0.0048 *
Drinkers					
CC	58	41	1.00 (ref)	1.00 (ref)	
CG	76	87	1.62 (0.98–2.68)	1.69 (0.97–2.54)	0.0801
GG	42	61	2.05 (1.17–3.60)	2.11 (1.24–3.52)	0.0168 *

^a^, by univariate logistic regression analysis; ^b^, by multivariate logistic regression analysis adjusting for confounding factors; *, statistically significant; CI, confidence interval; aOR, adjusted odds ratio.

## Data Availability

The genotyping results and clinical data supporting the findings of this study are available from the corresponding authors upon reasonable requests via email at artbau2@gmail.com.
